# Green oxidations: Titanium dioxide induced tandem oxidation coupling reactions

**DOI:** 10.3762/bjoc.5.24

**Published:** 2009-05-25

**Authors:** Vineet Jeena, Ross S Robinson

**Affiliations:** 1 Department of Chemistry, University of KwaZulu-Natal, Scottsville, Pietermaritzburg, 3209, South Africa

**Keywords:** quinoxalines, tandem oxidation process, titanium dioxide

## Abstract

The application of titanium dioxide as an oxidant in tandem oxidation type processes is described. Under microwave irradiation, quinoxalines have been synthesized in good yields from the corresponding α-hydroxyketones.

## Introduction

Titanium dioxide has found widespread industrial application ranging from whiteners in paint [1], and additives in food [2], to UV absorbers in sunscreen lotions [3]. Its popularity stems from its inertness, low cost, and chemical stability under irradiation [4]. In the laboratory, its utility has been extended to the photodegradation of pesticides [5] and carcinogenic dyes [6], to sterilization against bacteria [7]. From a synthetic chemistry point of view, titanium dioxide’s main use has been to oxidize alcohols to its corresponding carbonyl derivatives which has been reported many times [8–10]. Titanium dioxide has also been used to synthesize dihydropyrazines [11], piperazines [12], and quinoxalines [13] although in low yields.

**Scheme 1:**
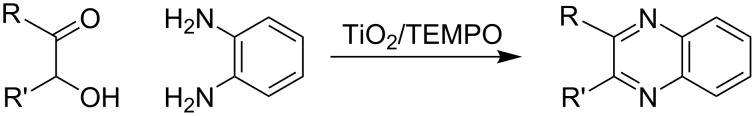
Titanium dioxide/TEMPO mediated synthesis of quinoxalines.

Herein, we describe the application of titanium dioxide in conjunction with 2,2,6,6-tetramethylpiperidine-1-oxyl radical (TEMPO) as an oxidant in the synthesis of quinoxalines, *via* the tandem oxidation process (TOP) (Scheme 1).

## Results and Discussion

Titanium dioxide in the anatase phase is a photocatalyst with a band gap of 3.2 eV corresponding to a wavelength of 387 nm [14]. Thus, we attempted to evaluate titanium dioxide as a potential TOP type catalyst based on the excellent work of Taylor and co-workers [15–16] by examining its behaviour under various energy sources (Table 1).

**Table 1:** Evaluation of energy sources.

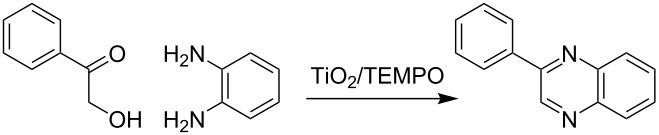			
Entry	Energy source	Time (h)	Yield (%)^a,b^

i	Natural sunlight	3	90
ii	Incandescent lamp	3	25
iii	UV light	3	10
iv	Microwave	10 min	87^c^

^a^Mixture of TiO_2_ (0.5 mmol), alcohol (0.5 mmol), amine (0.5 mmol) and TEMPO (0.05 mmol) in 3 ml methanol. ^b^Isolated yield. ^c^Absence of solvent.

Titanium dioxide is known to be highly reactive under sunlight [17] and this seemed to be the most obvious starting point. Initially, 2-hydroxyacetophenone was reacted with *o*-phenylenediamine under natural sunlight. We were delighted to observe the formation of the quinoxaline derivative in an isolated yield of 90% in 3 h. While encouraged by this result we immediately realized the limitations of this procedure. The use of an incandescent lamp gave a disappointing yield of 25% while a low wattage UV light afforded an equally disappointing yield of 10% in 3 h. We explored the use of microwave energy, which has been claimed to substantially improve reaction rates [18]. Surprisingly, the use of microwave energy produced the best result with the quinoxaline derivative isolated in a yield of 87% in 10 min. With the optimized procedure in hand, an investigation into the scope of the methodology, using a range of alcohols and diamines, was conducted (Table 2).

**Table 2:** Titanium dioxide catalysed tandem oxidation process under microwave irradiation.

Entry	α-Hydroxyketone	Diamine	Quinoxaline	Time (min)	Yield (%)^a^

i	**1a** 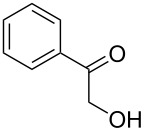	**2a** 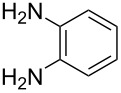	**3a** 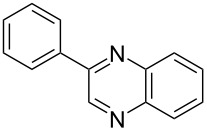	10	87
ii	**1b** 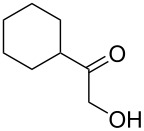	**2a** 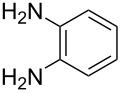	**3b** 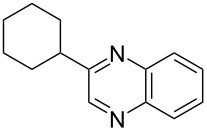	10	60
iii	**1c** 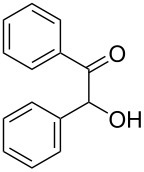	**2a** 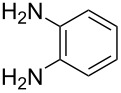	**3c** 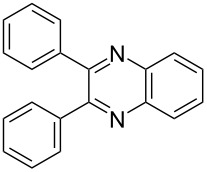	20	81
iv	**1d** 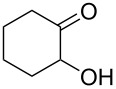	**2a** 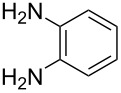	**3d** 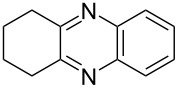	10	88
v	**1a** 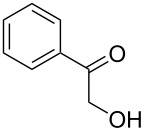	**2b** 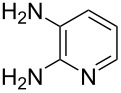	**3e** 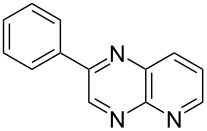	10	83^b^
vi	**1c** 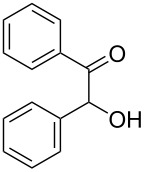	**2b** 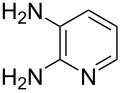	**3f** 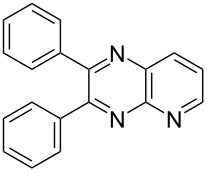	20	54
vii	**1d** 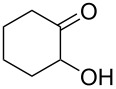	**2b** 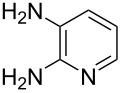	**3g** 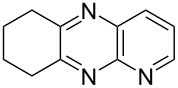	20	56

^a^Isolated yield. ^b^Isolated as a mixture of regioisomers.

After the successful synthesis of quinoxaline **3a**, the effect of an alkyl substituent was explored, which is often claimed to be problematic due to the ‘hyper-reactivity’ of the intermediate keto-aldehyde [19]. The reaction afforded the quinoxaline derivative **3b** in a satisfactory isolated yield of 60%. The coupling of secondary alcohols **1c** and **1d** both proceeded smoothly, even though the hindered alcohol **1c** required a longer reaction time. Finally, the diamine component was varied (entries v-vii) with satisfactory yields obtained for both primary and secondary alcohols.

Concerning the mechanism, when titanium dioxide is irradiated with an appropriate energy source, electrons are promoted from the valence band to the conduction band leaving behind positive holes in the valence band [20]. The positive holes and electrons migrate to the surface where the holes react with water (or bound hydroxyl groups) to produce hydroxyl radicals which are strong oxidants [21]. Nevertheless, the activation of titanium dioxide under microwave irradiation is surprising since UV light is more intense than microwave energy. However, low levels of hydroxyl radical formation have been reported when titanium dioxide was subjected to microwave irradiation [22]. It has been suggested [23] that microwave energy can couple with a crystalline solid generating a non-thermal distribution, resulting in an increase in ion mobility, which leads to the diffusion of electrons and positive holes to the surface and subsequent hydroxyl radical formation [24]. It is believed that a similar interaction is taking place in our system and in conjunction with TEMPO lead to the formation of keto-aldehydes or diketones, which are trapped in situ to produce the required products.

In an attempt to extend the methodology, a preliminary investigation into a titanium dioxide mediated tandem Wittig reaction was attempted in which the intermediate aldehyde is trapped by a stabilised phosphorane. Unfortunately, NMR spectroscopic analysis revealed a multitude of peaks, none of which were attributable to the product. It is believed that this is due to the non-selective nature of the hydroxyl radicals [25]. Currently studies are under way to moderate the reactivity of the titanium dioxide. In a recent publication [26], Zhao and co-workers reported the use of dye-sensitized titanium dioxide and TEMPO for the selective oxidation of alcohols to aldehydes and ketones. In our studies we have been examining similar systems with a view to incorporating them into tandem type processes.

## Conclusion

In summary, we have reported the use of titanium dioxide as a tandem oxidation catalyst, demonstrated by the rapid synthesis of quinoxalines under microwave irradiation. The process is advantageous due to the green credentials and low cost of the oxidant, short reaction times and good yields.

## Experimental

### 2-Phenylquinoxaline (**3a**)

2-Hydroxyacetophenone (0.068 g, 0.50 mmol), *o*-phenylenediamine (0.054 g, 0.50 mmol), titanium dioxide (0.040 g, 0.50 mmol) and TEMPO (0.008 g, 0.050 mmol) were added to a sealed 10 mL CEM Discover^®^ reaction vial equipped with a magnetic stirrer bar. The reaction vial was irradiated (at 150 W with cooling) for 10 min (2 × 5 min) at 150 °C, after which the vessel was rapidly cooled to 50 °C by the unit. The reaction mixture was diluted with dichloromethane (DCM) and passed through a short silica plug. The solvent was removed *in vacuo* to produce a crude product which was purified using radial chromatography (3:1 PE:EtOAc) to afford the pure product.

## Supporting Information

Supporting information features experimental procedures and spectroscopic analysis for compounds **3a**–**3g** coupling reactions.

File 1 Experimental and spectroscopic data for: Green oxidations: Titanium dioxide induced tandem oxidation
