# Supplementary material for: Green oxidations: Titanium dioxide induced tandem oxidation coupling reactions
Source: Beilstein J Org Chem. 2009 May 25;5:24. doi: 10.3762/bjoc.5.24 (PMC2707015; doi:10.3762/bjoc.5.24)
Supplement: File 1 — Experimental and spectroscopic data for: Green oxidations: Titanium dioxide induced tandem oxidation [file Beilstein_J_Org_Chem-05-24-s001.doc]

# Supporting Information

**Experimental and spectroscopic data for:**

Green oxidations: Titanium dioxide induced tandem oxidation coupling reactions

Vineet Jeena and Ross S. Robinson*

*Department of Chemistry, University of KwaZulu-Natal, Scottsville,*

*Pietermaritzburg, 3209, South Africa*

*robinsonr@ukzn.ac.za*

**General**

^1^H and ^13^C NMR spectra were obtained using a Bruker Avance 400 operating at either at 400 or 100 MHz using CDCl_3_ as an internal standard. Data are expressed in parts per million relative to residual solvent. All *J* values given in Hz. Low resolution electron impact (EI) mass spectra were recorded on Thermofinnigan trace GC, coupled with PolarisQ mass spectra. IR spectra were recorded on Perkin-Elmer Spectrum One. Absorption maxima are expressed in wavenumbers (cm^−1^). Radial chromatography was performed on the Harrison Research Chromatatron (Model 7924T) with the solvent system delivered by gravity flow using a 1 mm layer of Merck silica gel (7749).

The α-hydroxyketone **1b** was synthesized from the corresponding methyl ketone using the method described by Moriarty *et al*. [1] PE refers to petroleum ether with a boiling point range 40–60 °C.

**Microwave**

All microwave reactions were conducted on a CEM Focused Microwave^TM^ Synthesis system which uses an infrared sensor located below the microwave cavity floor to measure temperature.

**Temperature/Time Profile for 2-phenylquinoxaline (3a)**

**
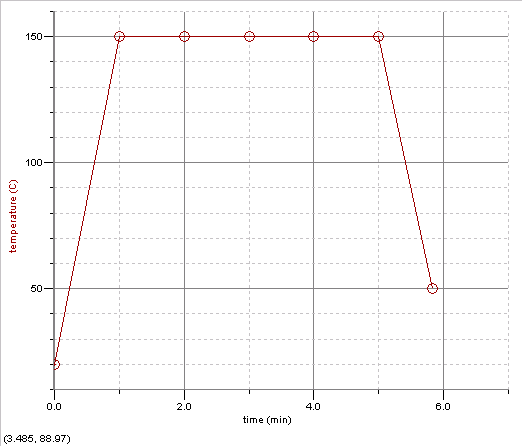
**

- Reaction temperature and profile was monitored every minute during a 5 min run.
- After the 5 min, the reaction vessel was rapidly cooled to 50 °C by the instrument.
- Once a temperature of 50 °C was reached, the instrument stopped cooling and released the residual pressure.

**General procedure for the synthesis of quinoxalines (table 2, entries i-vii)**

**2-Phenylquinoxaline (3a)** 2-Hydroxyacetophenone (0.068 g, 0.50 mmol), *o*-phenylenediamine (0.054 g, 0.50 mmol), TiO_2_ (0.040 g, 0.50 mmol) and 2,2,6,6-Tetramethylpiperidine-1-oxyl (TEMPO) (0.008 g, 0.050 mmol) were added to a sealed 10 mL CEM Discover® reaction vial equipped with a magnetic stirrer bar. The reaction vial was irradiated (at 150 W with cooling) for 10 min (2 x 5 min) at 150 °C, after which the vessel was rapidly cooled to 50 °C by the unit. The reaction mixture was diluted with dichloromethane (DCM) and passed through a short silica plug. The solvent was removed *in vacuo* to produce a crude product which was purified using radial chromatography (3 : 1 PE : EtOAc) to produce the title compound **3a** (0.089 g, 87%) as an orange solid: R_f_ 0.46 (3 : 1 PE : EtOAc); ν_max_ (neat) 1599, 1541, 1488, 1445, 1305 cm^−1^; ^1^H NMR (400 MHz, CDCl_3_) 7.53–7.58 (3H, m), 7.78–7.80 (2H, m), 8.16 (2H, m), 8.21 (m, 2H), 9.34 (1H, s); δ_C_ (100 MHz) 127.5, 127.6, 129.2, 129.6, 129.7, 130.2, 130.4, 136.8, 141.4, 142.4, 143.2, 151.9; m/z (EI) 206 (M^+^). Data consistent with literature [2].

**2-Cyclohexylquinoxaline (3b)** Prepared by the procedure given for **3a** using 1-cyclohexyl-2-hydroxyethanone **1b** [1] (0.106 g, 0.50 mmol) and *o*-phenylenediamine (0.054 g, 0.50 mmol). Purified using radial chromatography (2: 1 PE : EtOAc) to produce the title compound **3b** (0.064 g, 60%) as a brownish solid: R_f_ 0.80 (2 : 1 PE : EtOAc); *v*_max_ (neat) 1559, 1491, 1449, 1368 cm^−1^; ^1^H NMR (400 MHz, CDCl_3_) δ_H_ 1.38–2.07 (10H, m), 2.99 (1H, tt, *J =* 12.0 Hz *J =* 3.5 Hz), 7.72 (2H, m); 8.07 (2H, m); 8.78 (1H, s); δ_C_ (100 MHz) 25.9, 26.4, 32.3, 45.0, 128.9, 128.9, 129.1, 129.9, 141.4, 142.1, 144.9, 161.1 *m/z* (EI) 212 (M^+^). Data consistent with literature [2].

**2,3-Diphenylquinoxaline (3c)** Prepared using the procedure given for **3a** using benzoin (0.106 g, 0.50 mmol) and *o*-phenylenediamine (0.054 g, 0.50 mmol) but irradiated for 20 min. Purified using radial chromatography (9 : 1 PE : EtOAc) to give the title compound **3c** (0.114 g, 81%) as a yellow solid: R_f_ 0.59 (9 : 1 PE : EtOAc); *ν*_max_ (neat) 1596, 1514, 1448, 1346 cm^−1^; ^1^H NMR (400 MHz, CDCl_3_) δ_H_ 7.34–7.36 (6H, m), 7.52–7.54 (4H, m), 7.78 (2H, m), 8.19 (2H, m); δ_C_ (100 MHz) 128.2, 128.8, 129.2, 129.8, 129.9, 139.1, 141.2, 153.5; *m/z* (EI) 282 (M^+^). Data consistent with literature [2].

**1,2,3,4-Tetrahydrophenazine (3d)** Prepared using the procedure given for **3a** using 2-hydroxycyclohexanone dimer (0.057 g, 0.500 mmol) and *o*-phenylenediamine (0.054 g, 0.50 mmol). Purified using radial chromatography (1 : 1 PE : EtOAc) to give the title compound **3d** (0.810 g, 88%) as a white solid: R_f_ 0.54 (1 : 1 PE : EtOAc); *ν*_max_ (neat) 1459, 1423, 1384, 1330, 1291, 1238 cm^−1^; ^1^H NMR (400 MHz, CDCl_3_) δ_H_ 2.04 (4H, m), 3.16 (4H, m), 7.65 (2H, m), 7.97 (2H, m); δ_C_ (100 MHz) 22.8, 33.2, 128.3, 128.9, 141.2, 154.1; *m/z* 184 (M^+^). Data consistent with literature [3].

**2-/3-Phenylpyrido[2,3-*b*]pyrazine (3e)** Prepared using the procedure given for **3a** using 2-hydroxyacetophenone and 2,3-diaminopyridine **2b** (0.054g, 0.50 mmol) but irradiated for 20 min. Purified using radial chromatography (EtOAc) to give the title compound **3e** (0.087 g, 83%) as a brown solid, as a mixture of regioisomers with 3-phenylpyrido[2,3-*b*]pyrazine predominating: R_f_ 0.70 (EtOAc); *ν*_max_ (neat) 1645, 1561, 1457, 1419, 1363, 1327 cm^−1^; ^1^H NMR (400 MHz) 3-phenylpyrido[2,3-*b*]pyrazine δ_H_ 7.55–7.60 (3H, m), 7.69 (1H, dd, *J =* 8.3 Hz, *J =* 4.2 Hz), 8.32–8.35 (2H, m), 8.48 (1H, dd, *J =* 8.3 Hz, *J =* 1.8 Hz), 9.18 (1H, dd, *J =* 4.1 Hz, *J =* 1.8 Hz), 9.45 (1H, s); δ_C_ (100 MHz) 124.7, 128.0, 129.1, 131.0, 135.7, 136.7, 138.1, 144.3, 150.8, 153.4; 154.4. 2-phenylpyrido[2,3-*b*]pyrazine δ_H_ (400 MHz) 7.53–7.60 ( 3H, m), 7.73 (1H, dd, *J =* 8.4 Hz, *J =* 4.2 Hz), 8.21–8.24 (2H, m), 8.51 (1H, dd, *J =* 8.4 Hz *J =* 1.8 Hz), 9.17 (1H, dd *J =* 4.1 Hz *J =* 1.8 Hz), 9.55 (1H, s); δ_C_ (100 MHz) 125.5, 127.6, 129.2, 130.7, 135.8, 137.5, 138.5, 146.2, 150.4, 152.9, 154.6; *m/z* 207 (M^+^). Data consistent with literature [2].

**2,3-Diphenylpyrido[2,3-*b*]pyrazine (3f)** Prepared using the procedure given for **3a** using benzoin (0.106g, 0.50mmol) and 2,3-diaminopyridine (0.054 g, 0.50 mmol) but irradiated for 20 min. Purified using radial chromatography (9:1 DCM:EtOAc) to produce the title compound **3f** (0.076 g, 54%) as a yellow solid R_f_ 0.30 (9:1 DCM:EtOAc); ν_max_ (neat) 1588, 1545, 1430, 1382, 1328 cm^−1^; ^1^H NMR (400 MHz) δ_H_ 7.31–7.41 (6H, m), 7.56 (2H, d, *J =* 7.8 Hz), 7.64 (2H, d, *J =* 7.8 Hz), 7.74–7.76 (1H, dd, *J =* 8.4 Hz *J =* 4.1 Hz), 8.55–8.57 (1H, d, *J =* 8.2 Hz), 9.19 (1H, d, *J =* 4.2 Hz); δ_C_ (100 MHz) 125.2, 128.2, 128.5, 129.4, 129.6, 129.8, 130.3, 136.2, 138.0, 138.5, 149.4, 153.6, 155.0, 156.6; *m/z* 283 (M^+^). Data consistent with literature [4].

**6,7,8,9-Tetrahydropyrido[2,3-*b*]quinoxaline (3g)** Prepared using the procedure given for **3a** using 2-hydroxycyclohexanone dimer (0.057 g, 0.50 mmol), 2,3-diaminpyridine (0.054 g, 0.50 mmol) but irradiated for 20 min. Purified using radial chromatography (9:1 DCM: EtOAc) to produce the title compound **3g** (0.052 g, 56%) as a purple solid R_f_ 0.40 (9:1 DCM:EtOAc); *ν*_max_ (neat) 2926, 2858, 1459, 1425, 1380, 1327, 1261 cm^−1^; ^1^H NMR (400 MHz) δ_H_ 2.05 (4H, m), 3.22 (2H, m), 3.25 (2H, m), 7.59–7.62 (1H, dd, *J =* 8.3 Hz *J =* 4.2 Hz), 8.30–8.33 (1H, dd, *J =* 8.3 Hz *J =* 1.6 Hz), 9.05 (1H, dd, *J =* 3.9 Hz *J =* 1.5 Hz) δ_C_ (100 MHz) 22.4, 22.6, 33.1, 33.4, 124.3, 136.1, 137.2, 150.1, 152.8, 155.7, 157.9; *m/z* 185 (M^+^). Compound has been previously reported [5].

References:

1. Moriarty, R. M.; Berglund, B. A.; Penmasta, R. *Tetrahedron Lett.* **1992,** *33,* 6065–6068. [doi:10.1016/S0040-4039(00)60007-2](http://dx.doi.org/10.1016/S0040-4039(00)60007-2)
2. Raw, S. A.; Wilfred, C. D.; Taylor, R. J. K. *Org. Biomol. Chem*. **2004,** *2,* 788–796. [doi:10.1039/b315689c](http://dx.doi.org/10.1039/b315689c)
3. Petukhov, P. A.; Tkachev, A. V. *Tetrahedron*. **1997,** *53,* 9761–9768. [doi:10.1016/S0040-4020(97)00623-6](http://dx.doi.org/10.1016/S0040-4020(97)00623-6)
4. Mohsenzadeh, F.; Aghapoor, K.; Darabi, H. R. *J. Braz. Chem. Soc.* **2007,** *18,* 297–303. [doi:10.1590/S0103-50532007000200009.](http://dx.doi.org/10.1590/S0103-50532007000200009)
5. Antoniotti, S.; Duňach, E. *Tetrahedron Lett.* **2002,** *43,* 3971–3973. [doi:10.1016/S0040-4039(02)00715-3](http://dx.doi.org/10.1016/S0040-4039(02)00715-3)
